# Supplementary material for: Annals of Education: Teaching Climate Change and Global Public Health
Source: Int J Environ Res Public Health. 2023 Dec 27;21(1):41. doi: 10.3390/ijerph21010041 (PMC10815579; doi:10.3390/ijerph21010041)
Supplement: Supplementary file 1 [file ijerph-21-00041-s001.zip › ijerph-2732166-supplementary.pdf]

California Clean Energy Policy

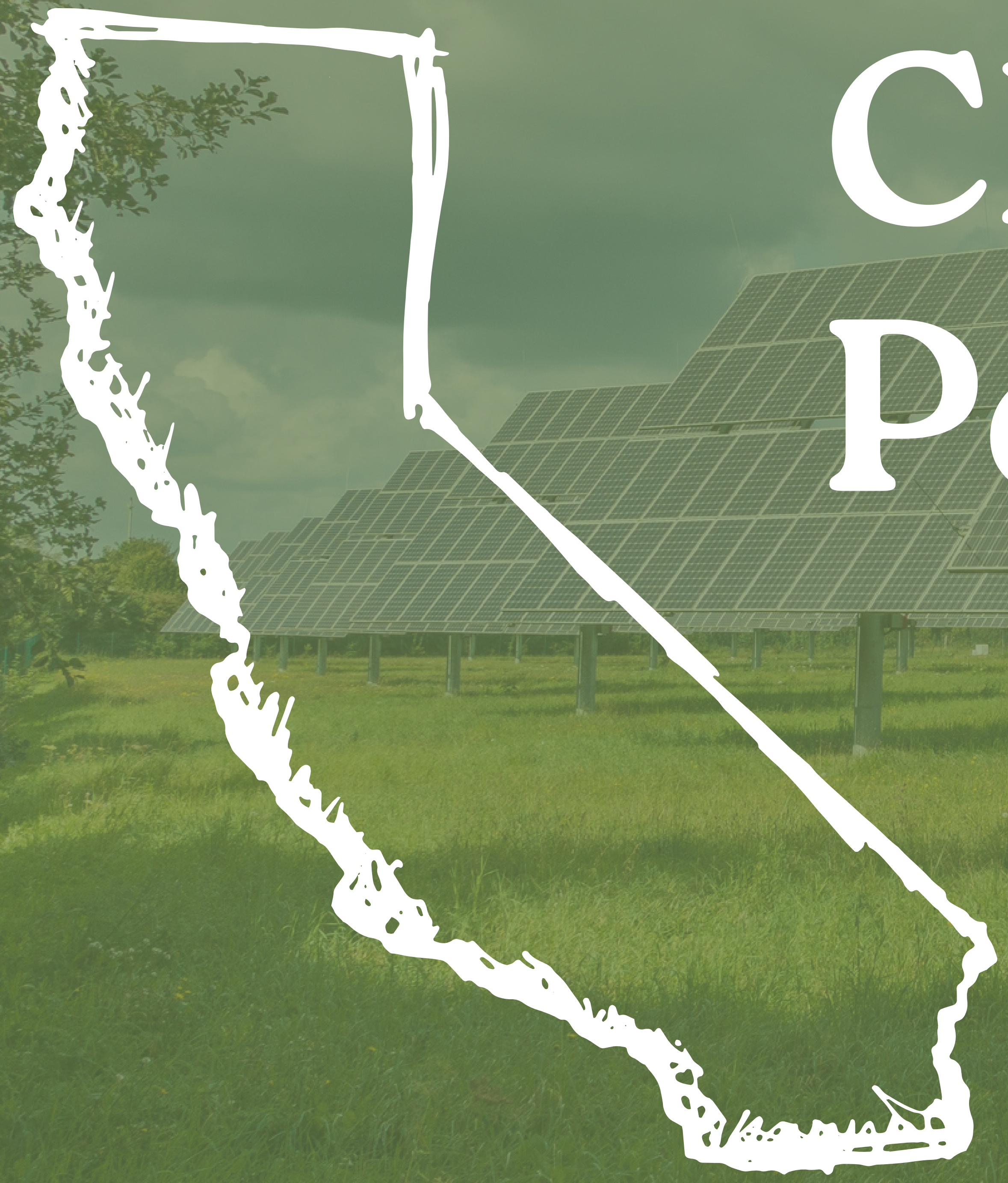

MARISA GUERRERO
NYU SCHOOL OF GLOBAL PUBLIC HEALTH
CLIMATE CHANGE & PUBLIC HEALTH
NOVEMBER 21, 2023

California: A Climate Leader

- **CA is the most populous state** in the US (39 million residents)
- **Largest economy** in the US, 5th largest globally
- **Progressive politics and strong public opinion on climate change** facilitate ambitious climate policy
  - Public opinion (Yale Climate Opinion Surveys, 2021):
    - 73% report being worried about global warming (+8 from national average)
    - 79% support funding research into renewable energy (+2)
    - 80% support providing tax rebates for energy-efficient vehicles and solar panels (+3)
- Energy usage and procurement
  - CA uses more energy than any other state, except TX
  - CA's per capita energy use is less than almost all other states, due to energy efficiency efforts
  - CA produces more electricity using renewable energy than every other state but TX

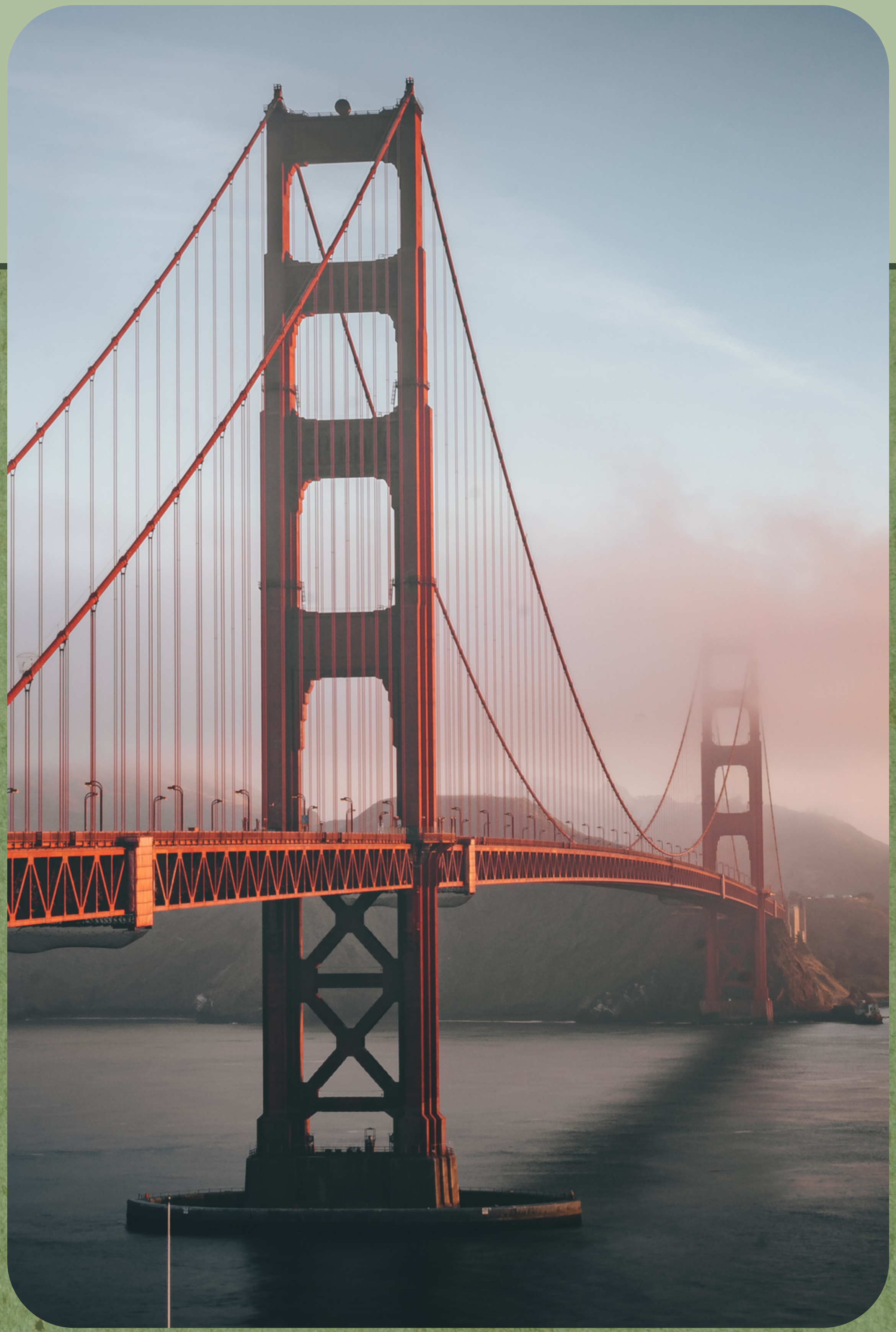

Clean Energy Goals & Key Policies

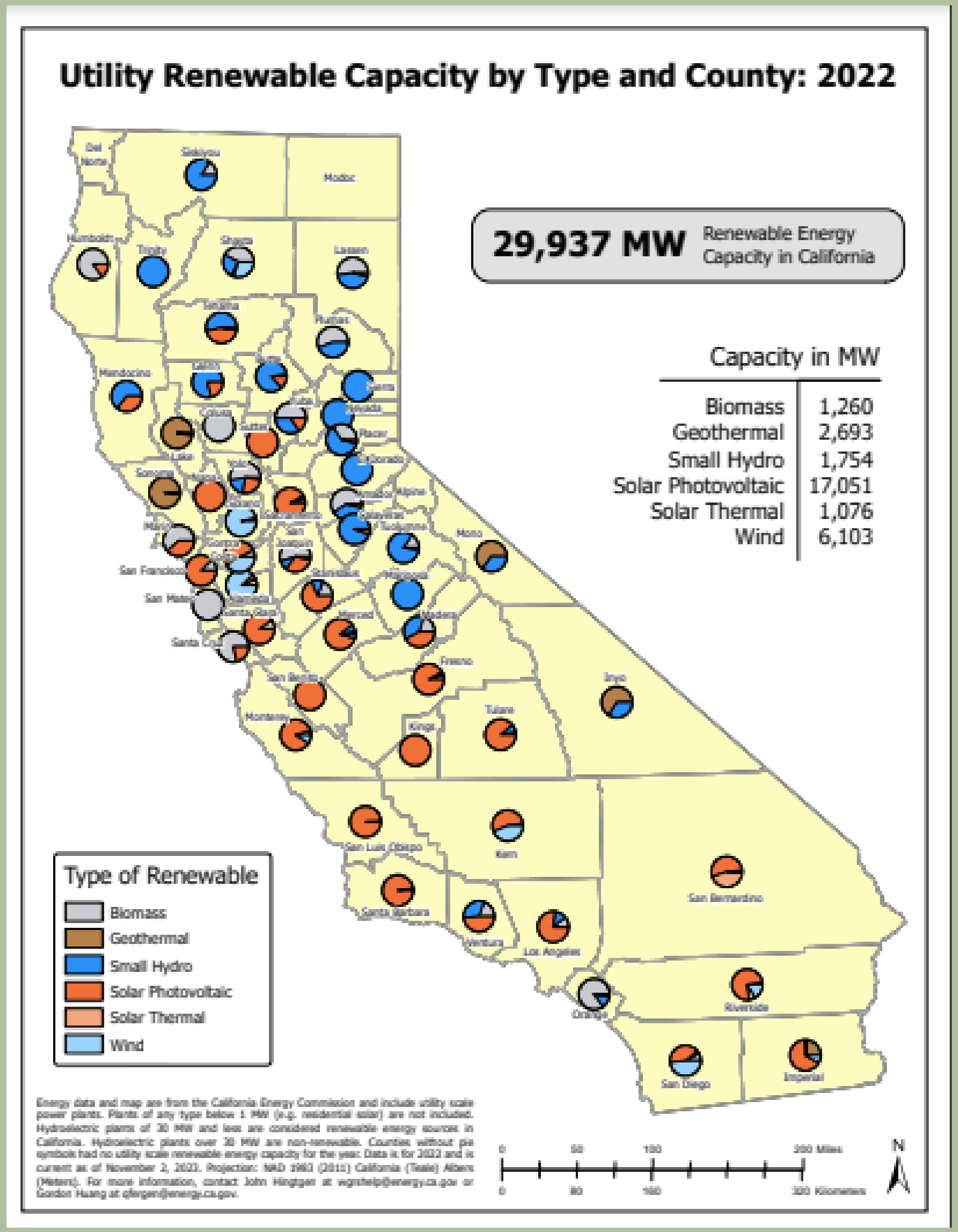

- **Renewables Portfolio Standard (2002+):** Requires electricity providers to procure a certain percentage of energy from renewable sources.
  - **100 Perfect Clean Energy Act (SB 100, 2018):** Sets an RPS goal to generate 60% of electricity from renewable energy sources by 2030 and **100% by 2045**.
- **CA Public Utilities Commission (2022)** set a goal to add 25.5 GW of renewable energy and 15 GW of storage by 2032.
- **CA Energy Commission (AB525, 2021)** established a preliminary planning goal of developing 2-5 GW of offshore wind by 2030 and 25 GW by 2045.
- **Western Grid Regionalization (2023):** Energy regulators from CA, WA, OR, AZ, NM announced a plan to form a new entity to manage power across states, which would further development of clean electricity sources and lower costs for consumers.
- **Funding from CA Energy Commission:** CEC provides grants and incentives to support clean energy development and use.

Solar

Goals:
100% zero-carbon electricity by 2045
(60% by 2030)

- **CA is the nation's top producer of electricity from solar energy**
  - In 2022, CA produced 31% of the nation's total utility-scale and small-scale solar PV electricity generation and 69% of U.S. utility-scale solar thermal electricity generation
- **Solar is the largest source of CA's renewable energy generation** - 27% of the state's net electricity generation in 2022 (19% from utility-scale, 8% from small-scale solar generation)
- **Current solar capacity:** At the beginning of 2023, CA had 32,000 MW of solar capacity
  - **Storage capacity:** 5600 MW of storage capacity (July 2023, a 10x increase since 2020)
- **Types of solar:**
  - **Solar thermal facilities + solar photovoltaic (PV) plants**
  - **Utility-Scale:** All CA solar thermal facilities and several of the largest solar PV plants are in CA's southeastern deserts
    - 7 utility-scale solar projects covering 19,000 acres of public land have been approved, and would provide 3,000 MW of electricity (powering 2 million homes)
  - **Small-Scale:** Solar systems with less than 1 MW capacity (aka rooftop solar)
- **Key CA solar policy:**
  - **2012 Western Solar Plan:** Blueprint for solar energy development in AZ, CA, CO, NV, NM, UT
  - **Community Solar (AB2316, 2022):** Creates a community renewable energy program under the CA Public Utilities Commission
  - **CA Solar Initiative (2006):** Includes financial incentives to install solar panels on homes

Wind

Goals:
(1) 100% zero-carbon electricity by 2045 (60% by 2030)
(2) Generate up to 5 GW from offshore wind by 2030 and 25 GW by 2045

- **CA is 8th in the nation in wind energy production**, which is projected to grow
  - Onshore + offshore wind accounted for 7% of CA's in-state electricity generation in 2022
- **Current wind capacity:** At the beginning of 2023, CA produced 6,200 MW of wind energy
- **Types of wind energy:**
  - **Onshore:** 6 major wind resource areas: Altamont, East San Diego County, Pacheco, Solano, San Gorgonio, Tehachapi
  - **Offshore:** CA has some of the best offshore wind resources in the country and is positioned to become a leader on floating offshore wind
- **Floating offshore wind developments**
  - Floating offshore wind suits the deep waters of the Pacific Outer Continental Shelf
  - Achieving 25 GW will require building 1,600 turbines (and will create 8,000 union jobs)
  - **5 new leases:** In 2022, the Bureau of Ocean Energy Management sold 5 leases for offshore wind projects in Morro and Humboldt Bays. Next step: set up a central procurement entity to buy the clean power generated by these projects, which will enable project financing.
- **Key CA wind policy:**
  - **AB525, 2021:** Requires CA Energy Commission to develop a strategic plan for offshore wind energy developments; sets offshore wind energy generation goals (25 GW/25m homes)

Geothermal

Goals:
100% zero-carbon electricity by 2045
(60% by 2030)

- **CA is the top producer of geothermal energy in the U.S.**
  - Geothermal power plants use steam to turn large turbines which run electrical generators
  - In 2022, CA produced 69% of the nation's utility-scale geothermal-sourced electricity
  - Geothermal power accounted for 6% of CA's utility-scale generation and 5% of the state's total in-state generation in 2022
- **Geothermal resource areas**
  - **Geysers in the Mayacamas Mountains north of San Francisco are the world's largest geothermal field and the largest complex of geothermal power plants** (725 MW of installed capacity)
  - **Other geothermal resources** in north-central volcanic areas, near the Salton Sea in Southern CA, and along the CA-NV border
- **Geothermal capacity:**
  - Operating geothermal plants have a combined total capacity of 1,867 MW (enough to power ~ 1 million homes)

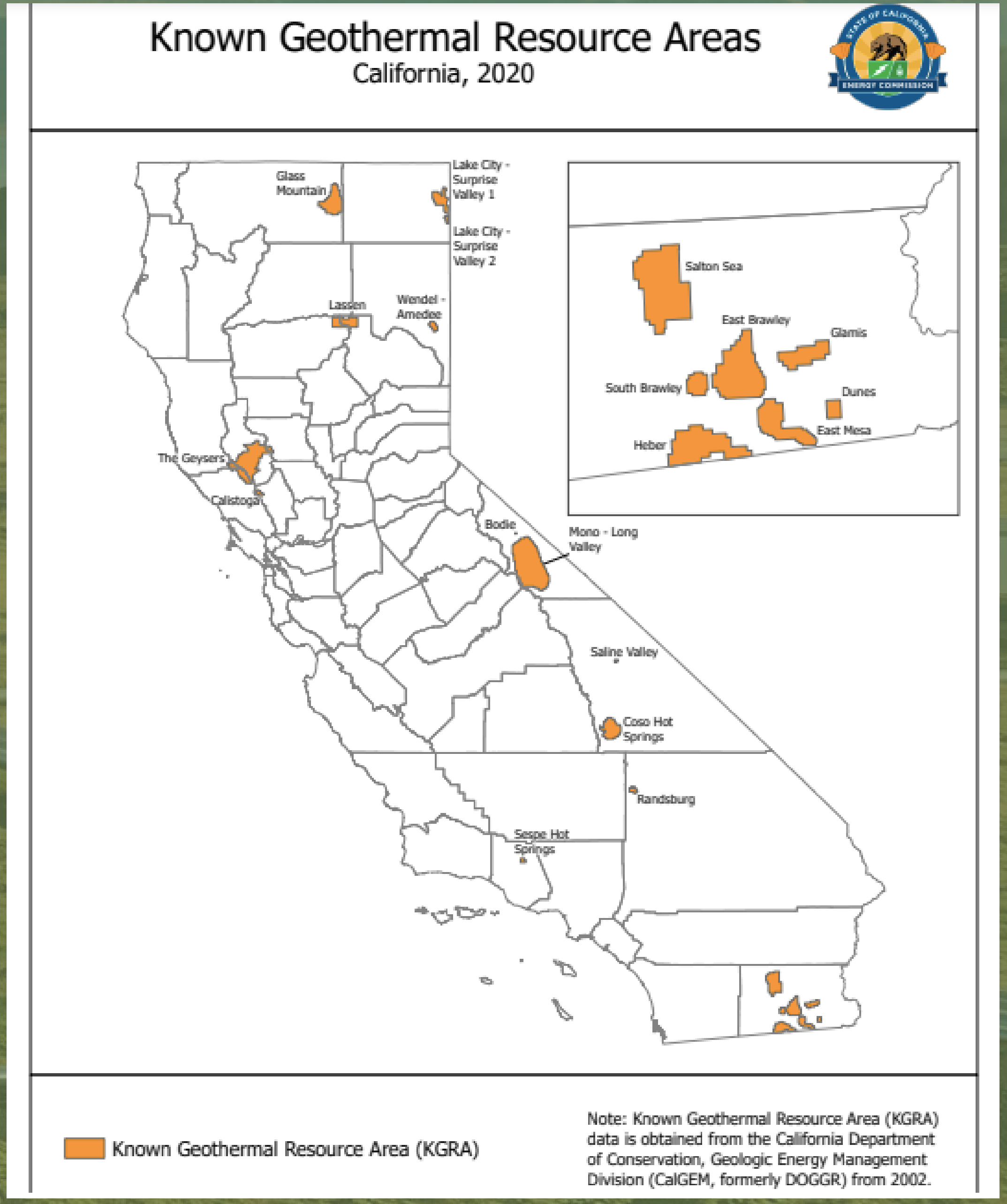

Hydroelectric

Goals:
100% zero-carbon electricity by 2045
(60% by 2030)

- **CA is consistently among the top four hydroelectric producers in the country** (hydroelectric capacity varies annual, dependent on rainfall)
  - **CA has the second-largest hydroelectric generating capacity** (after WA)
  - In 2022, hydroelectric accounted for 8% of CA in-state energy generation
- **Types of hydroelectric power:**
  - **Large hydro:** facilities larger than 30 MW
  - **Small hydro:** facilities smaller than 30 MW
- California's hydro generation plants are mostly in the eastern mountain ranges

Other California Climate Policies

REDUCING GREENHOUSE GAS EMISSIONS

CA Global Warming Solutions Act (AB32, 2006; SB32, 2016): Requires a reduction in GHG emissions to 1990 levels by 2020 and to 40% below 1990 levels by 2030, respectively.

DISINCENTIVIZING FOSSIL FUELS

- **Cap and Trade Program:** A system in which power plants, refineries, and other large facilities buy and sell GHG emissions allowances to meet overall emissions limitations set by CARB
- **GHG Reduction Fund:** State program allocating proceeds from cap-and-trade auctions to support investments and projects that reduce GHG emissions across the state
- **Climate Change Scoping Plan:** Comprehensive plan outlining how to achieve GHG emissions reductions targets

BUILDINGS SECTOR

- Commitment to 3 million climate-ready homes by 2030
- $1.4 billion to clean, healthy, and fossil free buildings
- **Green Building Standard Program (2022):** Energy efficiency standards and retrofits for existing buildings
- **Equitable Building Decarbonization Program (2022):** Commits $922 million over 4 years to installing all-electric appliances in low-income homes

TRANSPORTATION SECTOR

- **Automobile Emissions Standards (2022):** Sets first GHG emission standards for passenger vehicles
- **Electric Vehicle Charging (2015):** Requires local governments to plan EV charging infrastructure
- **Low Carbon Fuel Standard (2011):** Reduces the GHG emissions intensity in transportation fuel products
- **Advanced Clean Cars Program (2012, 2022):** Introduces vehicle performance standards and manufacturer requirements to reduce GHG and smog-causing pollutant emissions from cars

References

Baldassare, M. (2023). Fact Sheet: California Voter and Party Profiles. Public Policy Institute of California. https://www.ppic.org/publication/california-voter-and-party-profiles/

Berkeley Law. (2023). California Climate Policy Dashboard. https://www.law.berkeley.edu/research/clee/research/climate/climate-policy-dashboard

Borgeson, M. & Hassan, M. (2023). California Climate & Energy Policy Update: Summer 2023. Natural Resources Defense Council. https://www.nrdc.org/bio/merrian-borgeson/ca-climate-energy-policy-update-summer-2023

California Energy Commission. AB 525 Reports: Offshore Renewable Energy. https://www.energy.ca.gov/data-reports/reports/ab-525-reports-offshore-renewable-energy

California Energy Commission. (2023). Fact Sheet: California Offshore Wind. https://www.energy.ca.gov/sites/default/files/2023-11/California_Offshore_Wind_Fact_Sheet_ada.pdf

California Energy Commission. (2019). Fact Sheet: Developing Renewable Energy. https://www.energy.ca.gov/sites/default/files/2019-06/REN-DevelopingRenewableEnergy.pdf

California Energy Commission. Hydroelectric Power. https://www.energy.ca.gov/data-reports/california-power-generation-and-power-sources/hydroelectric-power

California Energy Commission. (2020). Map: Known Geothermal Resource Areas. https://cecgis-caenergy.opendata.arcgis.com/documents/CAEnergy::known-geothermal-resource-areas/explore

References

California Energy Commission. (2023). Map: Utility Renewable Capacity by Type and County: 2022. https://cecgis-caenergy.opendata.arcgis.com/documents/utility-renewable-capacity-by-type-and-county-2022/explore

California Energy Commission. Offshore Renewable Energy. https://www.energy.ca.gov/programs-and-topics/topics/renewable-energy/offshore-renewable-energy

California Energy Commission. Wind Energy in California. https://www.energy.ca.gov/data-reports/california-power-generation-and-power-sources/wind-energy-california

California Public Utilities Commission. (2023). CPUC Augments Historic Clean Energy Procurement Goals To Ensure Electric Reliability. https://www.cpuc.ca.gov/news-and-updates/all-news/cpuc-augments-historic-clean-energy-procurement-goals-to-ensure-electric-reliability-2023

Marlon, J. et al. (2022). Yale Climate Opinion Maps 2021. Yale Program on Climate Change Communication. https://climatecommunication.yale.edu/visualizations-data/ycom-us/

McGhee, H. et al. (2023). Fact Sheet: California's Population. Public Policy Institute of California. https://www.ppic.org/publication/californias-population/

Myskow, W. (2023). Solar Is Booming in the California Desert, if Water Issues Don't Get in the Way. Inside Climate News. https://insideclimatenews.org/news/26062023/solar-water-desert-center-california/

References

St. John, J. (2023). California ups renewables target again with new plan to add 85GW by 2035. Canary Media. https://www.canarymedia.com/articles/clean-energy/california-ups-renewables-target-again-with-new-plan-to-add-85gw-by-2035

Office of Governor Gavin Newsom. (2023). ICYMI: California Grid Reaches 5,600 MW of Battery Storage Capacity, a 1020% Increase Since 2020. https://www.gov.ca.gov/2023/07/12/icymi-california-grid-reaches-5600-mw-of-battery-storage-capacity-a-1020-increase-since-2020/

U.S. Department of the Interior. (2012). Obama Administration Approves Roadmap for Utility-Scale Solar Energy Development on Public Lands. https://www.doi.gov/news/pressreleases/Obama-Administration-Approves-Roadmap-for-Utility-Scale-Solar-Energy-Development-on-Public-Lands

U.S. Energy Information Administration. (2023). California State Profile and Energy Estimates. https://www.eia.gov/state/analysis.php?sid=CA

Velez, K. & Borgeson, M. (2022). CA Clean Buildings Progress Report: 2022. Natural Resources Defense Council. https://www.nrdc.org/bio/kiki-velez/ca-clean-buildings-progress-report-2022

Winkler, M. (2022). California Poised to Overtake Germany as World’s No. 4 Economy. Bloomberg. https://www.bloomberg.com/opinion/articles/2022-10-24/california-poised-to-overtake-germany-as-world-s-no-4-economy#xj4y7vzkg
